# The diversity of microfungi associated with grasses in the *Sporobolus indicus* complex in Queensland, Australia

**DOI:** 10.3389/ffunb.2022.956837

**Published:** 2022-08-19

**Authors:** Tracey V. Steinrucken, Joseph S. Vitelli, David G. Holdom, Yu Pei Tan

**Affiliations:** ^1^ Commonwealth Scientific and Industrial Research Organisation (CSIRO), Brisbane, QLD, Australia; ^2^ Department of Agriculture and Fisheries, Biosecurity Queensland, Brisbane, QLD, Australia; ^3^ Department of Agriculture and Fisheries, Plant Pathology Herbarium, Brisbane, QLD, Australia

**Keywords:** systematics, pathogen diversity, biological control, new taxa, grass endophytes, poaceae

## Abstract

There are five closely related *Sporobolus* species, collectively known as weedy *Sporobolus* grasses (WSG) or the rat’s tail grasses. They are fast growing, highly competitive, unpalatable weeds of pastures, roadsides and woodlands. An effective biological control agent would be a welcomed alternative to successive herbicide application and manual removal methods. This study describes the initial exploratory phase of isolating and identifying native Australian microfungi associated with WSG, prior to evaluating their efficacy as inundative biological control agents. Accurate species-level identification of plant-pathogenic microfungi associated with WSG is an essential first step in the evaluation and prioritisation of pathogenicity bioassays. Starting with more than 79 unique fungal morphotypes isolated from diseased *Sporobolus* grasses in Queensland, Australia, we employed multi-locus phylogenetic analyses to classify these isolates into 54 fungal taxa. These taxa belong to 22 Ascomycete families (12 orders), of which the majority fall within the Pleosporales (>24 taxa in 7 families). In the next phase of the study, the putative species identities of these taxa will allow us to prioritise those which are likely to be pathogenic based on existing literature and their known ecological roles. This study represents the first step in a systematic, high-throughput approach to finding potential plant pathogenic biological control agents.

## 1 Introduction

Five species of *Sporobolus* grasses (*S. africanus*, *S. fertilis*, *S. jacquemontii*, *S. natalensis*, and *S. pyramidalis*) are collectively recognised as the weedy *Sporobolus* grasses (WSG) or rat’s tail grasses across Australia (Palmer, 2012; Biosecurity Queensland, 2018). These five species, together with the native Australian species *S. blakei*, *S. creber*, *S. elongatus*, *S. laxus*, and *S. sessilis* (Simon and Jacobs, 1999; Peterson et al., 2014), belong to the *S. indicus* complex (Hetherington and Irwin, 1999; Peterson et al., 2017). The WSG spread prolifically, are unpalatable and lack nutrition for grazing livestock, which reduces carrying capacity for graziers and outcompetes native plants for resources (Palmer, 2012; Ansong et al., 2015). The WSG are morphologically similar and difficult to distinguish from closely related native *Sporobolus* species (Hetherington and Irwin, 1999; Biosecurity Queensland, 2018). This complicates management strategies, particularly the targeted application of herbicides. In addition, areas requiring WSG management are often vast and in remote locations. WSG are prime targets for biological control (biocontrol) solutions, either separately or in combination with herbicide application and manual control (Yobo et al., 2009; Lawrie, 2011; Lock, 2018; Sutton, 2019). Of the five WSG, *S. natalensis* is the primary target for control in Queensland as it impacts agriculture, pasture and biodiversity, from the New South Wales border to the Cape York Peninsula (Business Queensland, 2020).

Inundative biocontrol of WSG requires the formulation of a mycoherbicide from fungal pathogens endemic to Australia, which can be applied at a high dose causing an artificial, localised infection (Templeton and Greaves, 1984; Charudattan, 1988; McRae and Auld, 2000; Morin, 2020). Examples of successful mycoherbicides include *Colletotrichum gloeosporioides* to control *Aeschynomene virginica* (Northern joint vetch) (Templeton and Greaves, 1984) and *Nalanthamala diospyri* (syn. *Acremonium diospyri*) for *Diospyros virginiana* (persimmon trees) (Auld, 2000) in the USA. In Australia, DiBak® Parkinsonia is the only registered mycoherbicide that has successfully navigated the framework of the Australian Pesticide and Veterinary Medicine Authority (APVMA). DiBak® Parkinsonia contains three fungi (*Lasiodiplodia pseudotheobromae*, *Macrophomina phaseolina*, and *Neoscytalidium novaehollandiae*) for the biocontrol of the invasive legume *Parkinsonia aculeata* (Galea, 2021).

Host-range testing, virulence and pathogenicity trials, and safety evaluations are resource intensive but essential for the regulatory approval of biocontrol agents. Several fungi have previously been tested as potential biocontrol agents for WSG, but none have been shown to be suitable, either due to their efficacy as pathogens, or their lack of host specificity. The fungi previously tested include *Microdochium dawsoniorum*, *Neopestalotiopsis nebuloides*, and *Pestalotiopsis etonensis* (Lock, 2018; Kukuntod, 2020; and this study); *Nigrospora oryzae* (Lawrie, 2011; Fletcher and Leemon, 2015); four *Curvularia* species including *C. ravenelii* (Hetherington, 1997; Hetherington and Irwin, 1999); and *Ustilago sporoboli-indici* (Yobo et al., 2009; Palmer, 2012; Vitelli et al., 2017; Rapley, 2020).

Observations of WSG populations across several years of surveys at multiple locations in Queensland, revealed evidence of *S. natalensis* dieback or die-off, reduced fecundity of plants, lesions, and other symptoms of fungal disease (Vitelli et al., 2017). As a result of these and other surveys, 79 fungal isolates were collected from symptomatic tissues of *S. indicus* complex grasses, and transferred onto artificial medium, with the goal of testing them as potential biocontrol agents for the WSG. Many of the isolates in the collection are certainly novel and/or cryptic species, based on based on multi-locus sequence analysis. This is the first step in prioritising these fungi for testing as potential biocontrol agents for WSG.

## 2 Methods

### 2.1 Sample collection and fungal isolation

**Figure 1:**
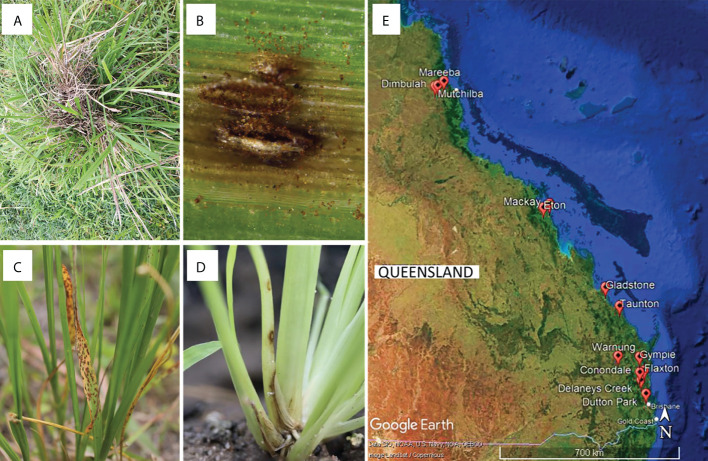
**(A–D)** Examples of disease symptoms on *Sporobolus* spp. Plants targeted for sampling, and **(E)** sampling sites for this study in Queensland, Australia generated using Google Earth Pro©.

Between early 2017 and mid-2021, plant tissue samples were collected from areas with infestations of WSG in Queensland (**Table S1**). Specifically, *S. indicus* complex grasses with disease symptoms (leaf chlorosis, leaf and stem lesions, and root death) were collected (**Figure 1**). Additional samples were collected from *Sporobolus* spp. cultivated in glasshouses at the Ecosciences Precinct, Dutton Park, QLD, Australia. A 5 cm sample of symptomatic plant material was surface sterilised by submersion in a solution of 70% v/v ethanol and 1% v/v sodium hypochlorite for 30 s, followed by 70% v/v ethanol, then rinsed twice in sterile distilled water, and dried in a laminar flow cabinet on sterile filter paper (as per Bills, 1996). Three to six leaf segments (~2 mm^2^) from each sample were placed on potato dextrose agar (PDA) amended with either streptomycin (sPDA; 50 mg L^−1^) or chloramphenicol (cPDA; 200 mg L^-1^) and incubated in the dark at room temperature (23−25 °C). As mycelia developed, isolates were sub-cultured onto fresh cPDA, followed by PDA plates for growth of pure cultures. Reference isolates generated in this study were deposited in the culture collection at the Queensland Plant Pathology Herbarium (BRIP), Dutton Park, QLD, Australia.

### 2.2 DNA extraction, amplification, and sequencing

Genomic DNA was extracted with either the Isolate II Plant DNA Kit (Bioline) or the ZymoBIOMICS DNA Miniprep Kit (Zymo Research) as per the manufacturers’ instructions, from ~100 mg of mycelium scraped from agar plates. Oligonucleotide primers and Polymerase Chain Reaction (PCR) conditions used to amplify and sequence the targeted loci are listed in the **Supplementary Material Table S2**. PCRs were performed either with the MyTaq DNA Polymerase (Bioline) or with Phusion HF Master Mix (New England Biolab) according to manufacturer’s instructions, using 10 μmol of each primer and 2−3 μL neat DNA extract, to a total reaction volume of 25 μL per reaction. PCR products were purified with the ISOLATE II PCR and Gel Kit (Bioline) according to manufacturer’s instructions, eluted with sterile distilled H_2_O, and submitted to Macrogen Inc. (Seoul, South Korea) for bidirectional sanger sequencing.

### 2.3 Phylogenetic analysis

DNA sequence chromatograms of the sequenced loci were viewed, edited and assembled in Geneious Prime v. 11.1.2 (Biomatters Ltd., Auckland, New Zealand) and deposited in GenBank (**Supplementary Material Table S1**). DNA sequences were aligned with selected reference sequences downloaded from NCBI (**Supplementary Material S3**) using the MAFFT algorithm (Katoh et al., 2002) as implemented in Geneious. An initial two-loci phylogeny was constructed with a combined alignment of the internal transcribed spacer (ITS) region and 28S large subunit ribosomal RNA (LSU) sequences with *Ustilago abaconensis* ex-type CBS 8380 as the outgroup. Based on this initial identification, a more in-depth phylogenetic analysis was undertaken for each family based on DNA sequences from additional nuclear loci including the 18S small subunit ribosomal RNA (SSU), partial region of the glyceraldehyde-3-phosphate dehydrogenase (*gapdh*), RNA-directed polymerase II subunit 2 (*rpb2*), beta-tubulin (*tub2*), and translation elongation factor 1-α (*tef1-α*). All phylogenies were constructed using maximum likelihood (ML) with the RAxML v. 7.2.8 (Stamatakis, 2014) plug-in in Geneious starting from a random tree topology. The nucleotide substitution model used was General Time-Reversible (GTR) with a gamma-distributed rate variation. In addition, the Bayesian analysis was performed using the MrBayes v.3.2.1 (Huelsenbeck and Ronquist, 2001) plug-in in Geneious. To remove the need for a priori model testing, the Markov chain Monte Carlo (MCMC) analysis was set to sample across the entire GTR model space with a gamma-distributed rate variation across the nucleotide sites. Ten million random trees were generated using the MCMC procedure with four chains. The sample frequency was set at 2000 and the temperature of the heated chain was 0.1. “Burn-in” was set at 25%, after which the log-likelihood values were stationary.

## 3 Results

### 3.1 Phylogenetic analysis

**Figure 2:**
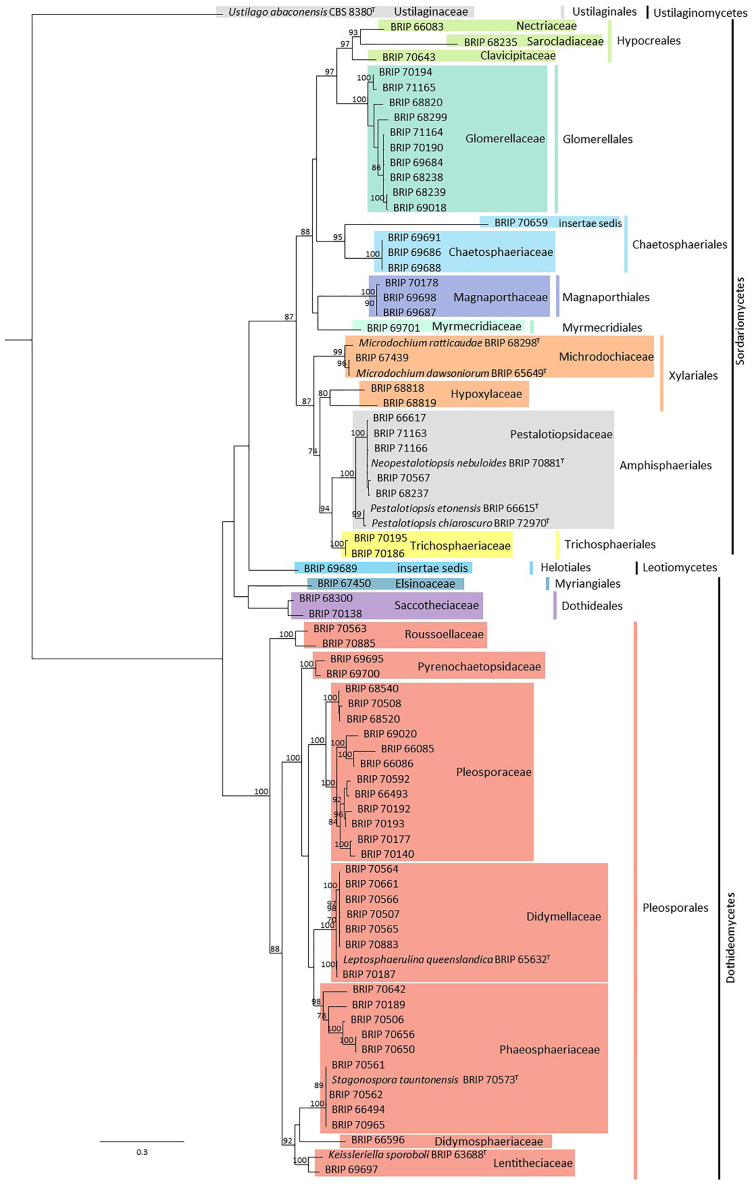
Phylogenetic tree inferred from a RAxML analysis based on a concatenated alignment of ITS and LSU sequences of the isolates in this study. RAxML bootstrap (bs) values greater than 70% are given at the nodes. In bold font are the isolates from this study and ex-type strains are indicated with ^T^.

In total, 79 fungal isolates were analysed in this study (**Supplementary Material Table S1**). Sequences from ITS and LSU resolved these isolates into 22 families representing 12 orders, all within the Ascomycota (**Figure 2**). Subsequent phylogenies used loci that were informative for each family.

#### 3.1.1 Amphisphaeriales

**Figure 3:**
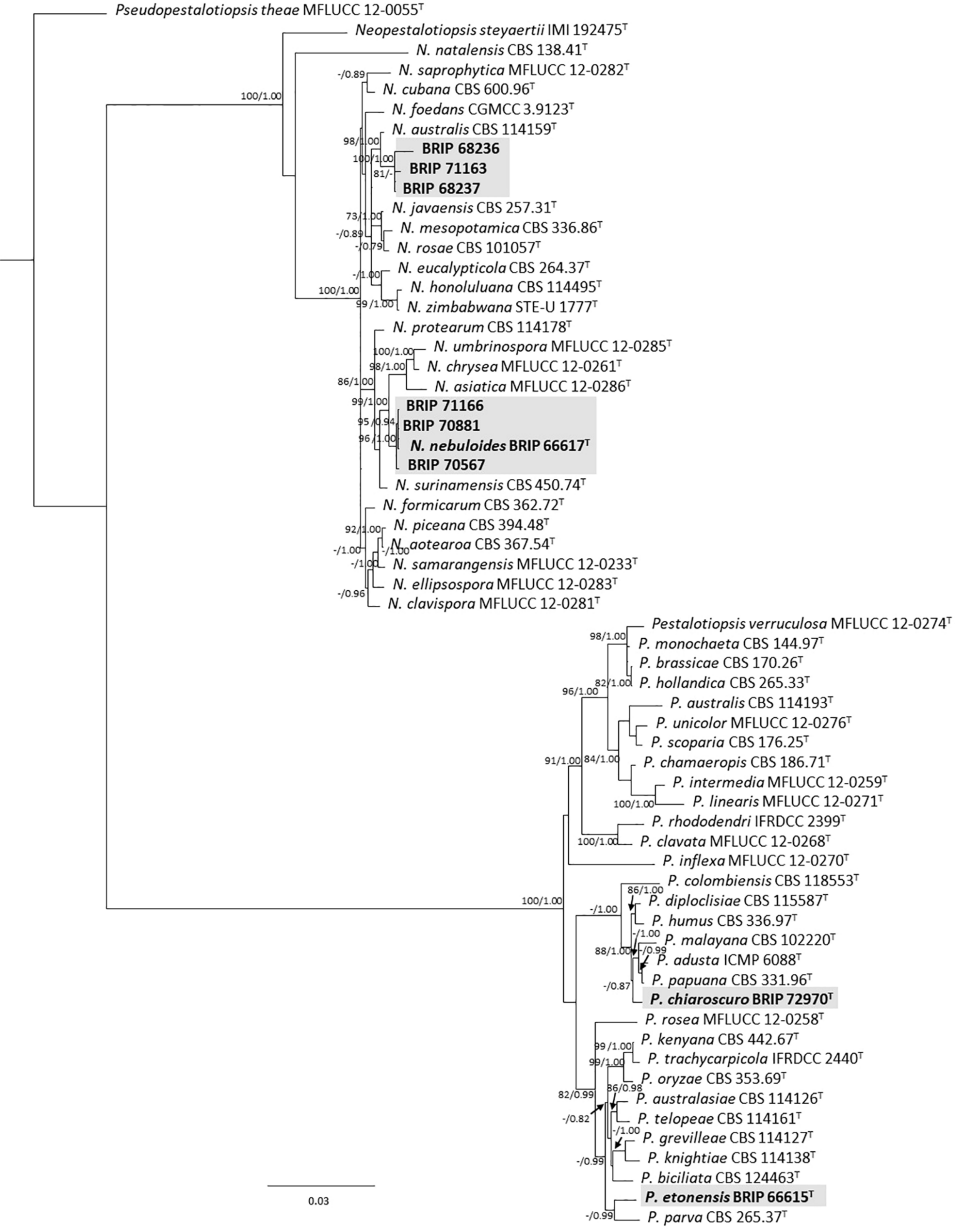
Phylogenetic tree inferred from a RAxML analysis based on a concatenated alignment of ITS, LSU, *tef1-α* and *tub2* sequences of related genera from Pestalotopsidaceae (Amphisphaeriales). RAxML bootstrap (bs) values greater than 70% and Bayesian posterior probabilities (pp) greater than 0.8 are given at the nodes (bs/pp). In bold font are the isolates from this study, and ex-type strains are indicated with ^T^.

The phylogenetic analysis of four gene regions (ITS, LSU, *tef1-α*, and *tub2*) resolved seven isolates into *Neopestalotiopsis* and *Pestalotiopsis* (Pestalotiopsidaceae; **Figure 3**). Three isolates (BRIP 70567, BRIP 70881, and BRIP 71166) were identified as *N. nebuloides*, a species described from *S. natalensis* (Crous et al., 2020), and three isolates (BRIP 68236, BRIP 68237, and BRIP 71163) are likely a novel *Neopestalotiopsis* species. One isolate (BRIP 72970) represented the holotype of *P. chiaroscuro* (Crous et al., 2022).

#### 3.1.2 Chaetosphaeriales

**Figure 4:**
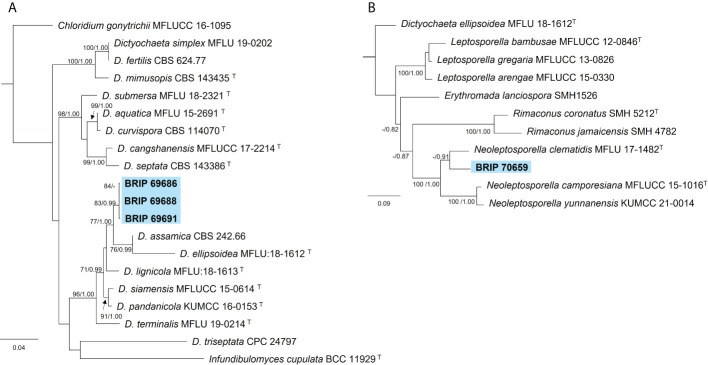
Phylogenetic trees inferred from RAxML analyses of strains in Chaetosphaeriales based on concatenated alignments of **(A)** ITS, LSU and *tef1-α* sequences of related species in Chaetosphaeriaceae; and **(B)** ITS and LSU sequences of related species in *Neoleptoporella* (*incertae sedis*). RAxML bootstrap (bs) values greater than 70% and Bayesian posterior probabilities (pp) greater than 0.8 are given at the nodes (bs/pp). In bold font are the isolates from this study, and ex-type strains are indicated with ^T^.

The phylogenetic analysis of three gene regions (ITS, LSU, and *tef1-α*) resolved four isolates into *Dictyochaeta* and *Neoleptosporella* (**Figure 4**). Three isolates isolated from *S. natalensis* roots were identified as phylogenetically close to *Dictyochaeta assimica* (Chaeotosphaeriaceae) (**Figure 4A**). Another isolate (BRIP 70659) is an undescribed species of *Neoleptosporella* (Chaetosphaeriales; **Figure 4B**). *Neoleptosporella* currently consists of three species associated with *Clematis* (Ranunculaceae; Phukhamsakda et al., 2020).

#### 3.1.3 Dothideales

**Figure 5:**
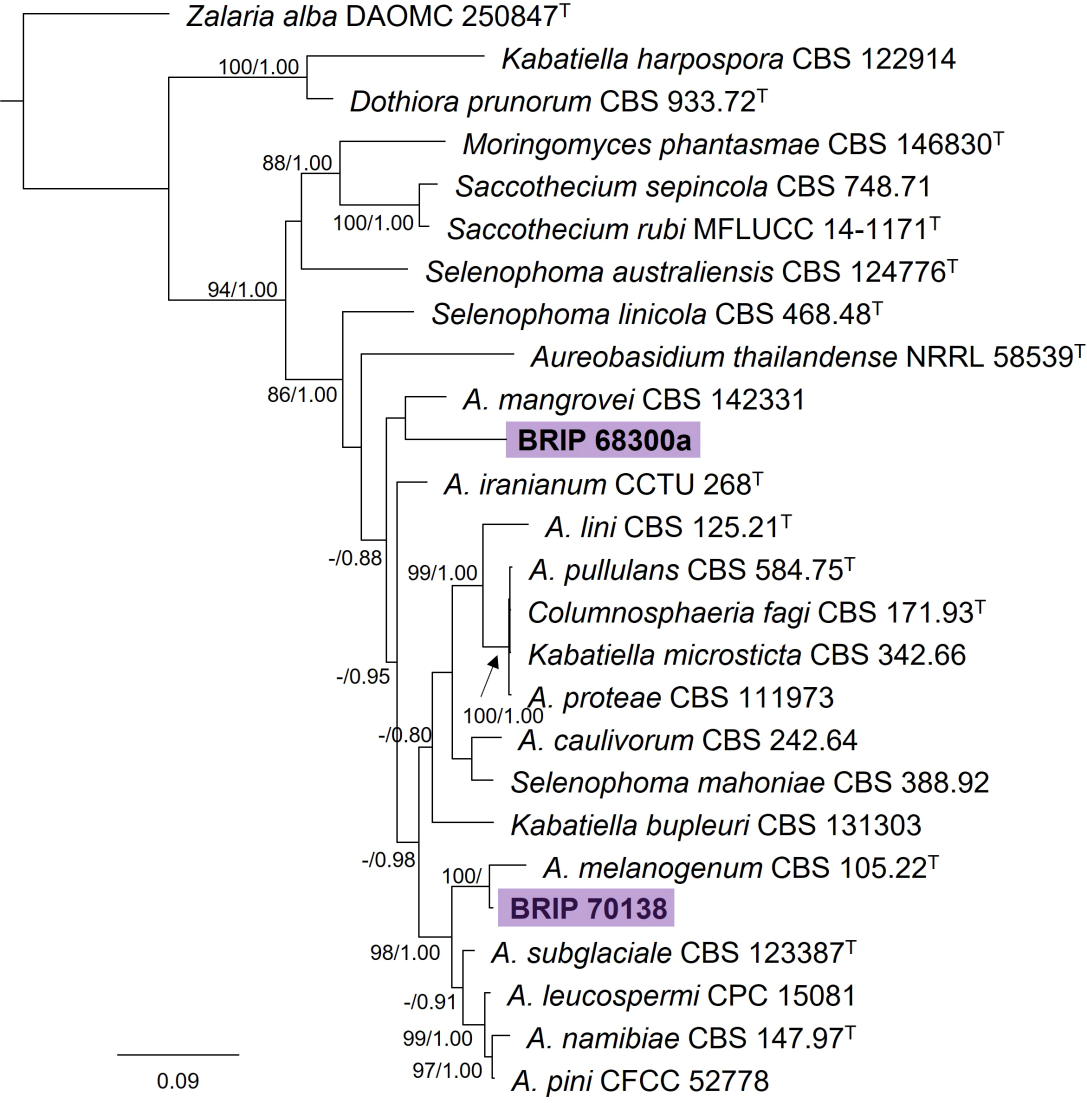
Phylogenetic tree inferred from a RAxML analysis based on a concatenated alignment of ITS, LSU, *rpb2* and *tub2* sequences of related genera in Saccotheciaceae (Dothideales). RAxML bootstrap (bs) values greater than 70% and Bayesian posterior probabilities (pp) greater than 0.8 are given at the nodes (bs/pp). In bold font are the isolates from this study, and ex-type strains are indicated by ^T^.

The phylogenetic analysis of four gene regions (ITS, LSU, *rpb2*, and *tub2*) identified two isolates as *Aureobasidium* (Saccotheciaceae; **Figure 5**). One isolate (BRIP 70138) was isolated from the seed of *S. natalensis* and is closely related to the ubiquitous *A. melanogeum*. The other isolate (BRIP 68300) was isolated from leaf tissue of *S. natalensis*, and is a novel species sister to *A. mangrovei*, a species described from plant debris in a freshwater habitat in Oman (Alaraimi et al., 2019).

#### 3.1.4 Glomerellales

**Figure 6:**
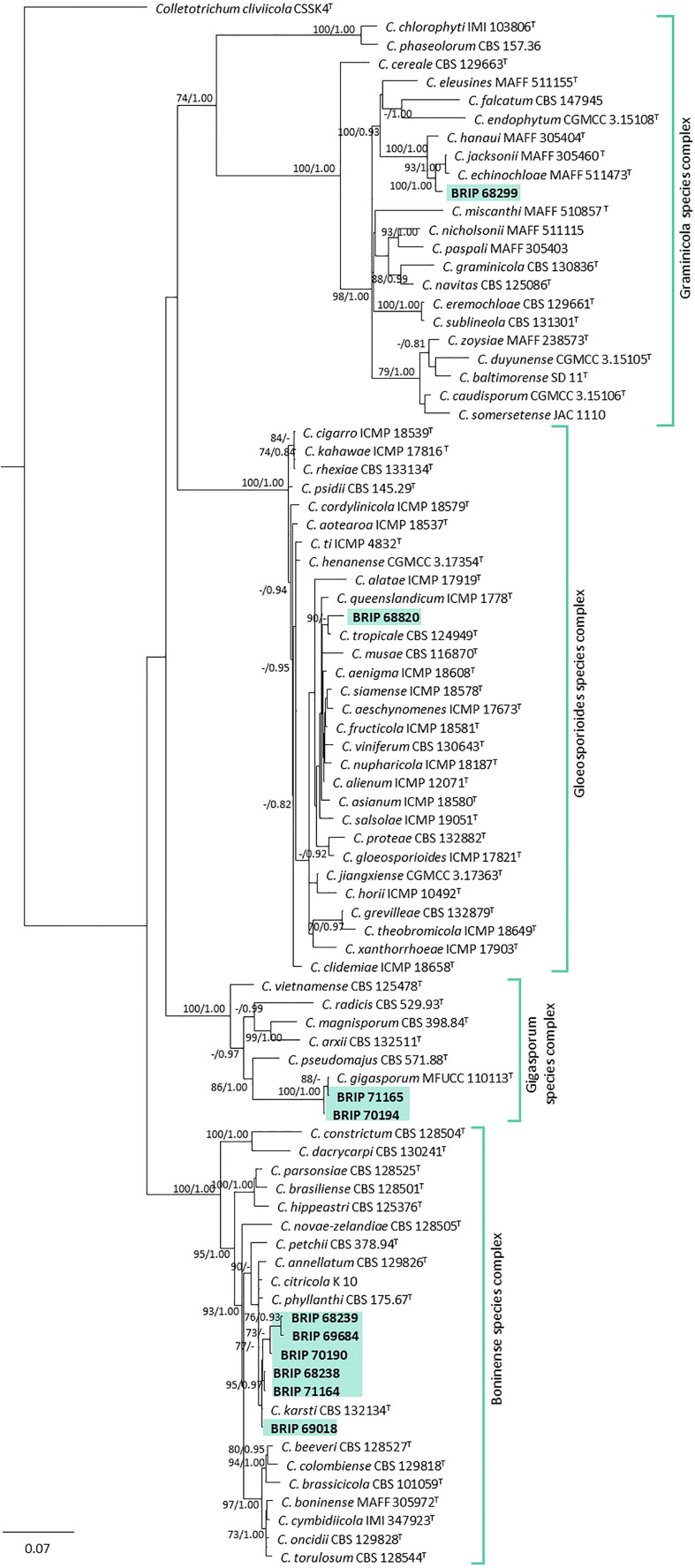
Phylogenetic tree inferred from a RAxML analysis based on a concatenated alignment of ITS, *tub2* and *gapdh* sequences of related genera from the *Colletotrichumgleosporioides*, *Co. graminicola*, and *Co. boninense* species complexes in Glomerellaceae (Glomerellales). RAxML bootstrap (bs) values greater than 70% and Bayesian posterior probabilities (pp) greater than 0.8 are given at the nodes (bs/pp). In bold font are the isolates from this study, and ex-type strains are indicated by ^T^.

The phylogenetic analysis of three gene regions (ITS, *gapdh*, and *tub2*) identified ten isolates as *Colletotrichum* (Glomerellaceae; **Figure 6**). Six of these isolates (BRIP 68238, BRIP 68239, BRIP 69018, BRIP 69684, and BRIP 70190) clustered in the same clade as the ex-type strain of *Co. karsti*, whilst two isolates (BRIP 70194, and BRIP 71165) clustered in the same clade as the ex-type strain of *Co. gigasporum*. Two isolates are likely to represent taxonomic novelties, one (BRIP 68820) in the *Co. gloeosporioides* species complex, and the other (BRIP 68299) in the *Co. graminicola* species complex.

#### 3.1.5 Helotiales

**Figure 7:**
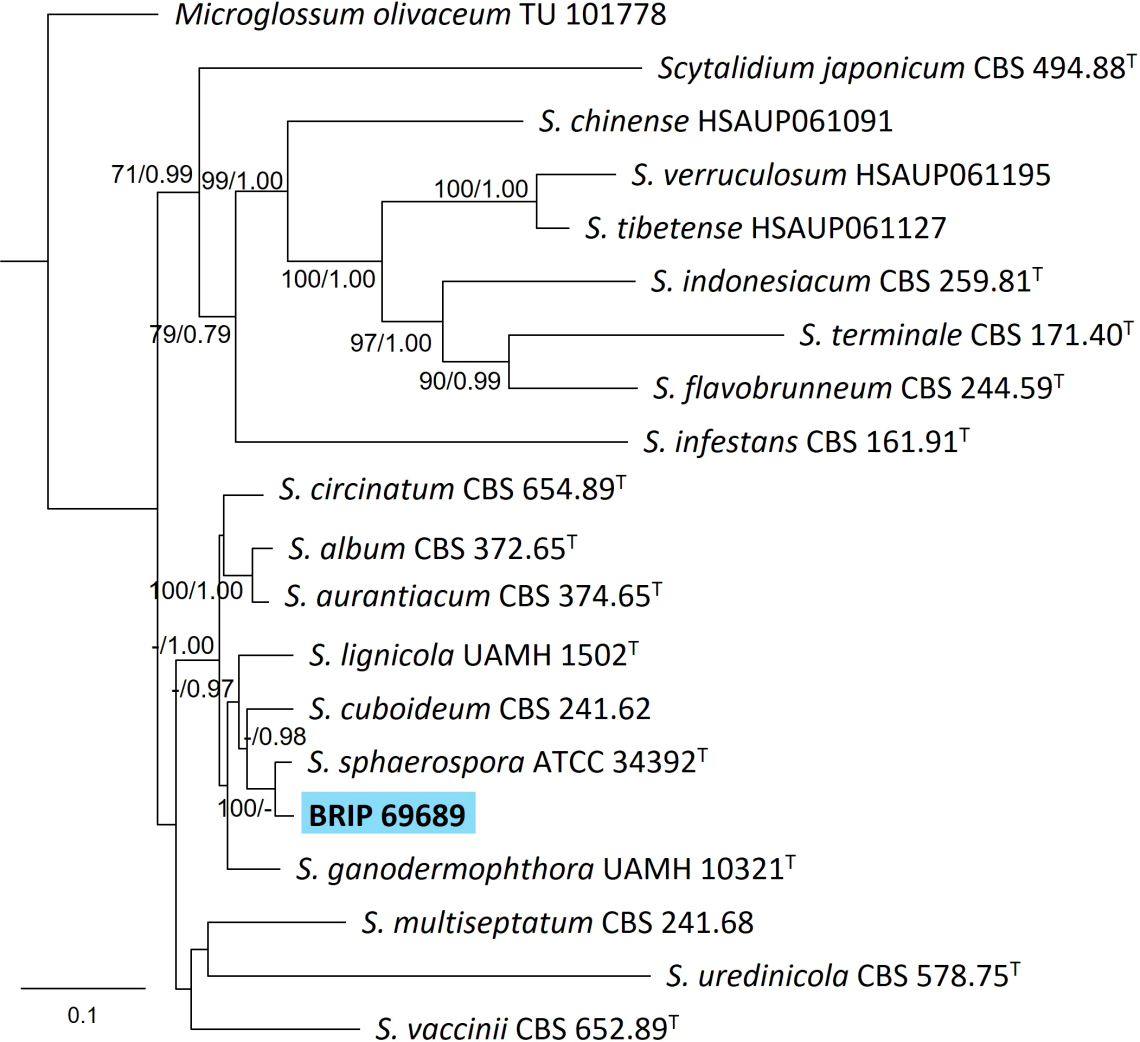
Phylogenetic tree inferred from a RAxML analysis based on a concatenated alignment of ITS, LSU, *rpb2* and SSU sequences of related genera from *Scytalidium* (Helotiales). RAxML bootstrap (bs) values greater than 70% and Bayesian posterior probabilities (pp) greater than 0.8 are given at the nodes (bs/pp). In bold font is the isolate from this study, and ex-type strains are indicated with ^T^.

The phylogenetic analysis of four gene regions (ITS, LSU, SSU, and *rpb2*) identified one isolate (BRIP 69689) isolated from the roots of *S. natalensis* as sister to the yellow rot fungus *Scytalidium sphaerosporum* (Helotiales; **Figure 7**).

#### 3.1.6 Hypocreales

**Figure 8:**
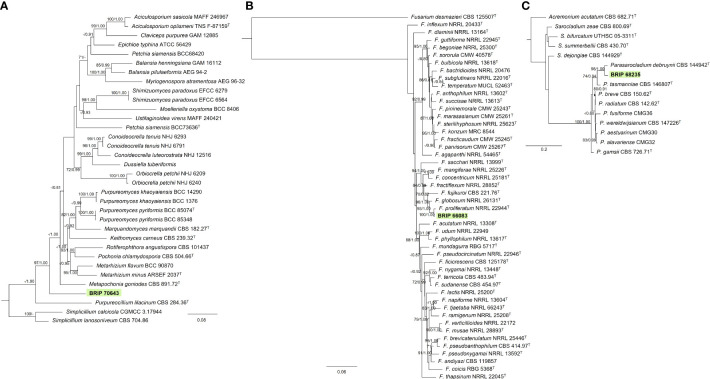
Phylogenetic trees inferred from RAxML analyses of strains in Hypocreales based on concatenated alignments of **(A)** *rpb2*, *tef1-α* and LSU sequences of related genera from Clavicipitaceae; **(B)** ITS, LSU, *rpb2*, *tef1-α* and *tub2* sequences of related species from the *Fusarium fujikuroi* species complex (Nectriaceae); and **(C)** ITS, LSU, *tub2* sequences of related genera from Sarocladiaceae (Hypocreales). RAxML bootstrap (bs) values greater than 70% and Bayesian posterior probabilities (pp) greater than 0.8 are given at the nodes (bs/pp). In bold font is the isolate from this study, and ex-type strains are indicated with ^T^.

Three isolates isolated from the leaves of *S. natalensis* were identified to belong to three families within Hypocreales. The phylogenetic analysis of three gene regions (LSU, *rpb2*, and *tef1-α*) identified one isolate (BRIP 70643) as a novel genus within Clavicipitaceae (**Figure 8A**). The phylogenetic analysis of four genes regions (ITS, LSU, *rpb2*, and *tef1-α*) identified one isolate (BRIP 66083) in the same clade as the ex-type strain of *Fusarium proliferatum* (Nectriaceae; **Figure 8B**). The phylogenetic analysis of three gene regions (ITS, LSU, and *tub2*) identified isolate BRIP 68235 as a novel species in *Parasarcocladium* (Sarocladiaceae; **Figure 8C**).

#### 3.1.7 Myriangiales

**Figure 9:**
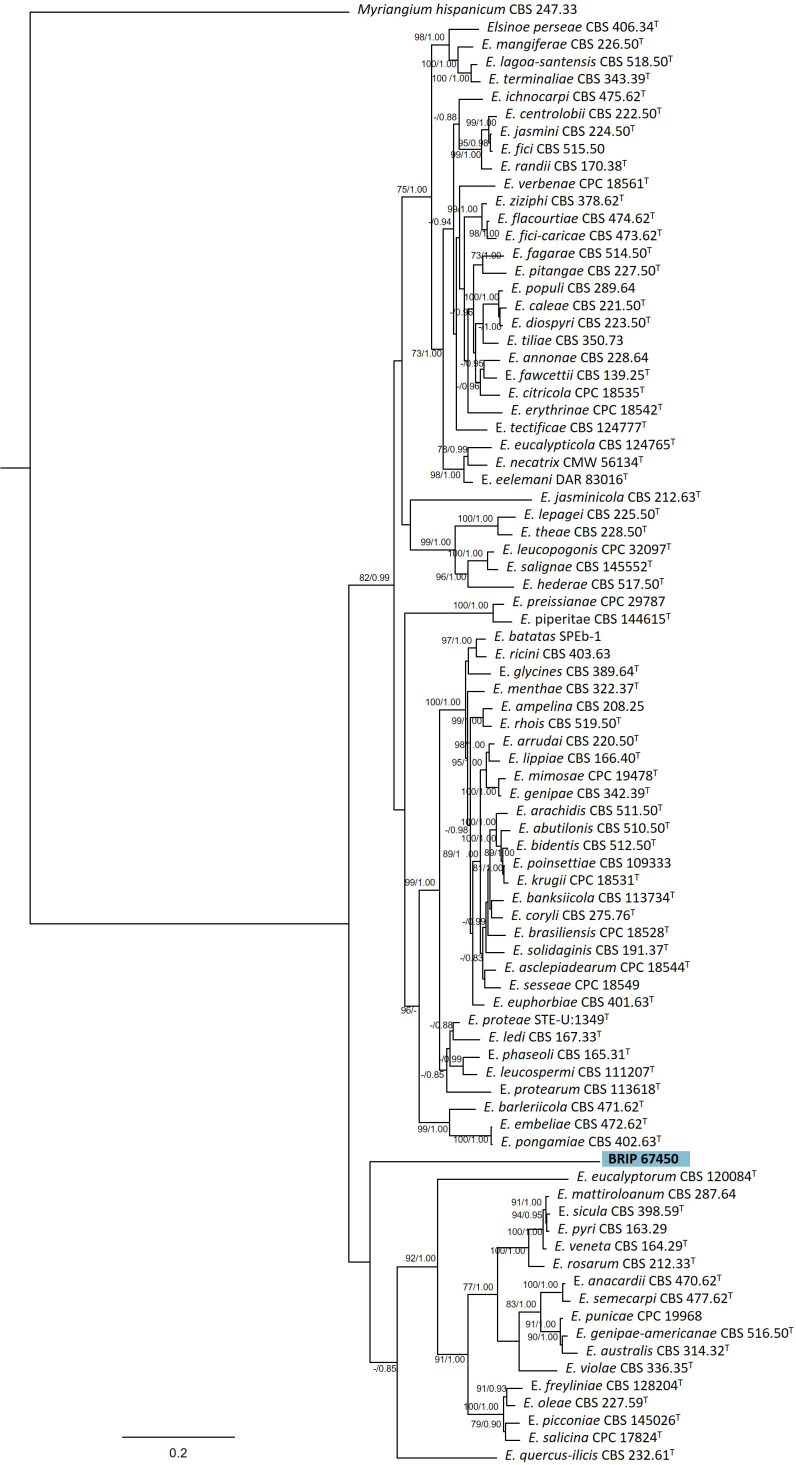
Phylogenetic tree inferred from a RAxML analysis based on a concatenated alignment of ITS, LSU, *rpb2* and *tef1*-α sequences of related genera from Elsinoaceae (Myriangiales). RAxML bootstrap (bs) values greater than 70% and Bayesian posterior probabilities (pp) greater than 0.8 are given at the nodes (bs/pp). In bold font is the isolate from this study, and ex-type strains are indicated with ^T^.

The phylogenetic analysis of four genes regions (ITS, LSU, *rpb2*, and *tef1-α*) identified one isolate (BRIP 67450) as a novel species in *Elsinoë* (Elsinoaceae; **Figure 9**).

#### 3.1.8 Pleosporales

**Figure 10:**
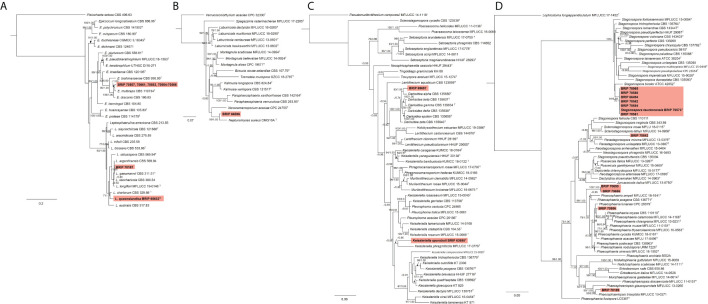
Phylogenetic trees inferred from RAxML analyses of strains in Pleosporales based on concatenated alignments of **(A)** ITS and *rpb2* sequences of related genera from Didymellaceae; **(B)** ITS, LSU, SSU and *tub2* sequences of related genera from Didymosphaeriaceae; **(C)** ITS, LSU and *tef1*-α sequences of related genera from Lentitheciaceae; and **(D)** ITS, LSU, SSU and *tef1-α* sequences of related genera from *Phaeosphaeria*ceae. RAxML bootstrap (bs) values greater than 70% and Bayesian posterior probabilities (pp) greater than 0.8 are given at the nodes (bs/pp). In bold font are the isolates from this study, and ex-type strains are indicated with ^T^.

Most of the isolates examined in this study belonged to six families within Pleosporales. The phylogenetic analysis of two gene regions (ITS and *rpb2*) identified three isolates to belong to Didymellaceae (**Figure 10A**). One species (BRIP 70507, BRIP 70661, BRIP 70883, and BRIPs 70564-70566) is a novel *Epicoccum*, isolated from stem, roots and leaves of *S. elongatus* and *S. natalensis*. This species was identified as closely related to *E. multiceps*. One isolate (BRIP 70187) was phylogenetically close to *Leptospherulina argentinensis* and *L. gaeumannii*. Another isolate (BRIP 65632) was identified as *L. queenslandica* (Crous et al., 2022).

The phylogenetic analysis of four gene regions (ITS, LSU, SSU, and *tub2*) identified one isolate (BRIP 66596) as a novel species in *Neptunomyces* (Didymosphaeriaceae; **Figure 10B**).

Two isolates to belonged to Lentitheciaceae based on three gene regions (ITS, LSU, and *tef1-α*; **Figure 10C**). One isolate (BRIP 63688) was identified as *Keissleriella sporoboli* (Crous et al., 2022), and another isolate (BRIP 69697) represents a novel *Darksidea*.

The phylogenetic analysis of four gene regions (ITS, LSU, SSU, *tef1-a*) resolved 11 isolates into five species within *Phaeosphaeria*ceae (**Figure 10D**). Three isolates (BRIP 70506, BRIP 70650, and BRIP 70656) represent two novel species in *Phaeosphaeria*. One isolate (BRIP 70642) is a novel species in *Parastagonospora*, and another isolate (BRIP 70189) is a novel species in *Phaeosphaeriopsis*. Interestingly, during this study, 30 isolates representing *Stagonospora tauntonensis* (Crous et al., 2022) were isolated from multiple *Sporobolus* spp. collected across the state of Queensland. Six of these isolates were included in the phylogeny.

**Figure 11:**
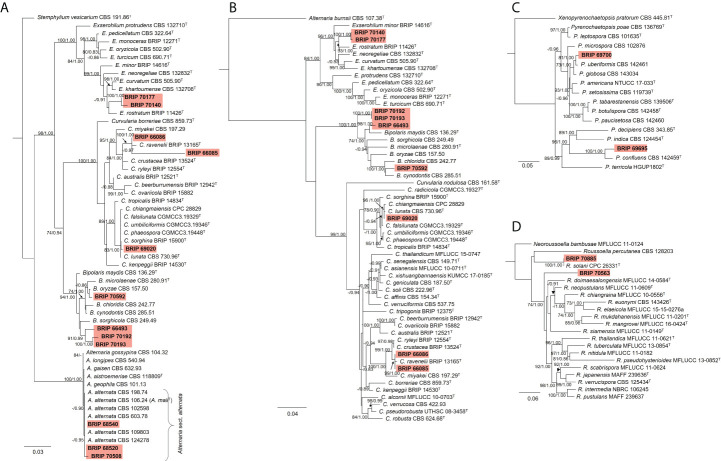
Phylogenetic trees inferred from RAxML analyses of strains in Pleosporales based on concatenated alignments of **(A)** ITS and LSU sequences of related species in *Alternaria*, *Bipolaris*, *Curvularia*, and *Exserohilum* (Pleosporaceae); **(B)** *gapdh*, *rpb2* and *tef1-α* sequences of related species in the *Bipolaris*, *Curvularia*, and *Exserohilum* (Pleosporaceae); **(C)** LSU, ITS, *rpb2* and *tub2* sequences of related species in Pyrenochaetopsidaceae; and **(D)** ITS, LSU, *rpb2* and *tef1-α* sequences of related species from Roussoellaceae. RAxML bootstrap (bs) values greater than 70% and Bayesian posterior probabilities (pp) greater than 0.8 are given at the nodes (bs/pp). In bold font are the isolates from this study, and ex-type strains are indicated with ^T^.

The phylogenetic analysis of the rDNA (ITS and LSU) found 12 isolates belonged to Pleosporaceae (**Figure 11A**). Three isolates (BRIP 68520, BRIP 68540, and BRIP 70508) clustered within *Alternaria* sect. *alternata*. Additional phylogenetic analysis based on three gene regions (*gapdh*, *rpb2*, and *tef1-α*) resolved the other nine Pleosporeaceae isolates into three genera, *Bipolaris*, *Curvularia*, and *Exserohilum* (**Figure 11B**). Two isolates (BRIP 70140 and BRIP 70177) represent a novel *Exserohilum* species. One isolate (BRIP 69020) is phylogenetically closely related to *Cu. lunata*, and two isolates (BRIP 66085 and BRIP 66086) are phylogenetically close to *Cu. ravenelii*. *Curvularia ravenelii* had been previously shown to infect *S. indicus* (Luttrell, 1976). It was assessed, but ultimately rejected, as a biocontrol agent against WSG in Australia (Hetherington and Irwin, 1999).

The phylogenetic analysis of four gene regions (LSU, ITS, *rpb2*, and *tub2*) identified two isolates (BRIP 69695 and BRIP 69700) as a novel species in *Pyrenochaetopsis* (Pyrenochaetopsidaceae; **Figure 11C**). The phylogenetic analysis of four gene regions (LSU, ITS, *rpb2*, and *tef1-α*) found two isolates belong to *Roussoella* (Roussoellaceae; **Figure 11D**). One isolate (BRIP 70885) was identified as *R. solani*, while the other isolate (BRIP 70563) is a novel species.

#### 3.1.9 Magnaporthales

**Figure 12:**
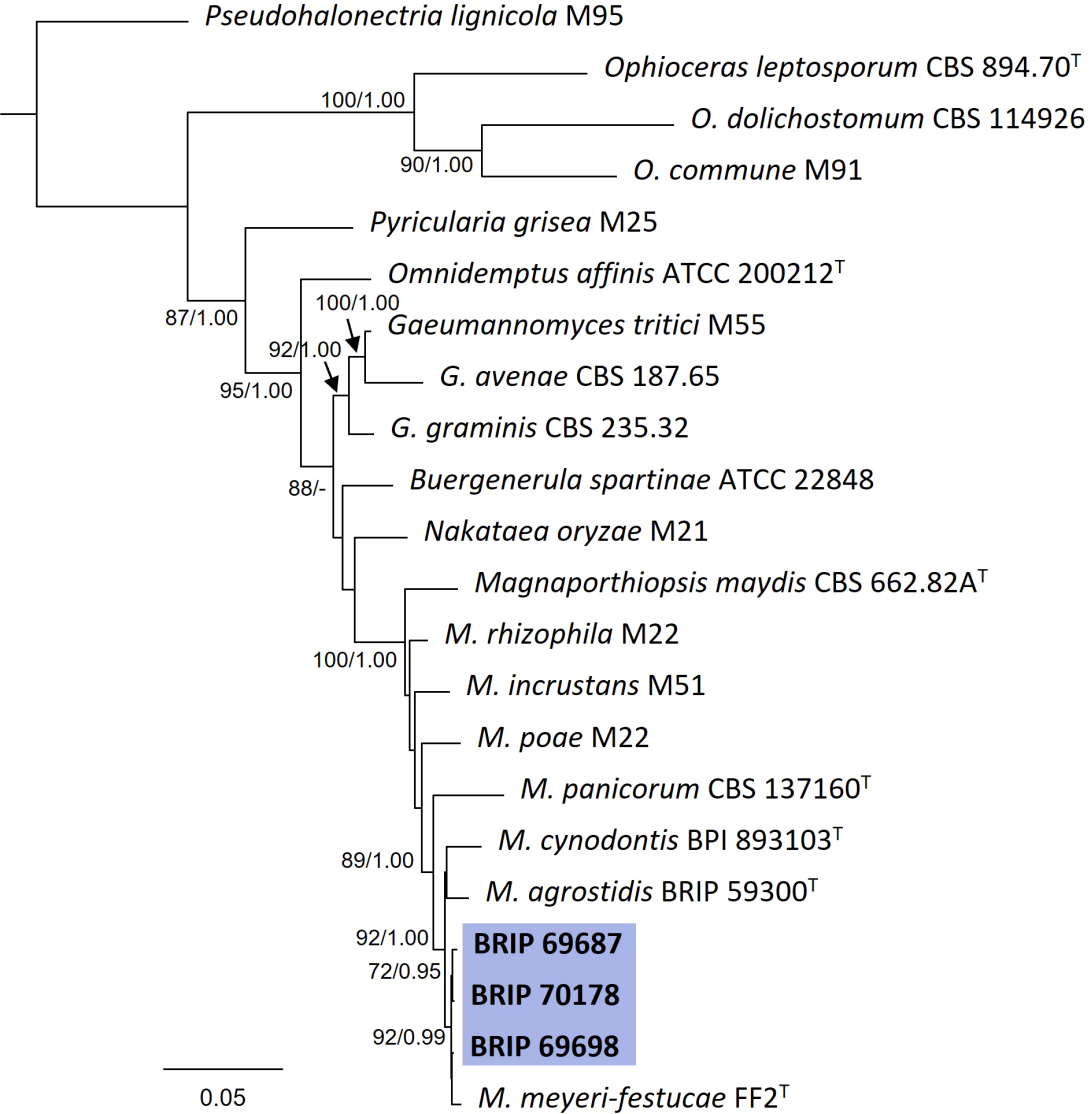
Phylogenetic tree inferred from a RAxML analysis based on a concatenated alignment of ITS, LSU, SSU and *tef1-α* sequences of related genera from Magnaporthaceae (Magnaporthiales). RAxML bootstrap (bs) values greater than 70% and Bayesian posterior probabilities (pp) greater than 0.8 are given at the nodes (bs/pp). In bold font are the isolates from this study, and ex-type strains are indicated with ^T^.

The phylogenetic analysis of four gene regions (ITS, LSU, SSU, and *tef1-α*) identified three isolates (BRIP 69687, BRIP 69698, and BRIP 70178) as Magnaporthiopsis meyeri-festucae (**Figure 12**). *Magnaporthiopsis meyeri-festucae* was associated with summer patch-like disease symptoms in *Festuca* spp. (Poaceae) in the USA (Luo et al., 2017). This study represents a new host record for *M. meyeri-festucae*, and the first report in Australia (Wong et al., 2022).

#### 3.1.10 Myrmecridiales

**Figure 13:**
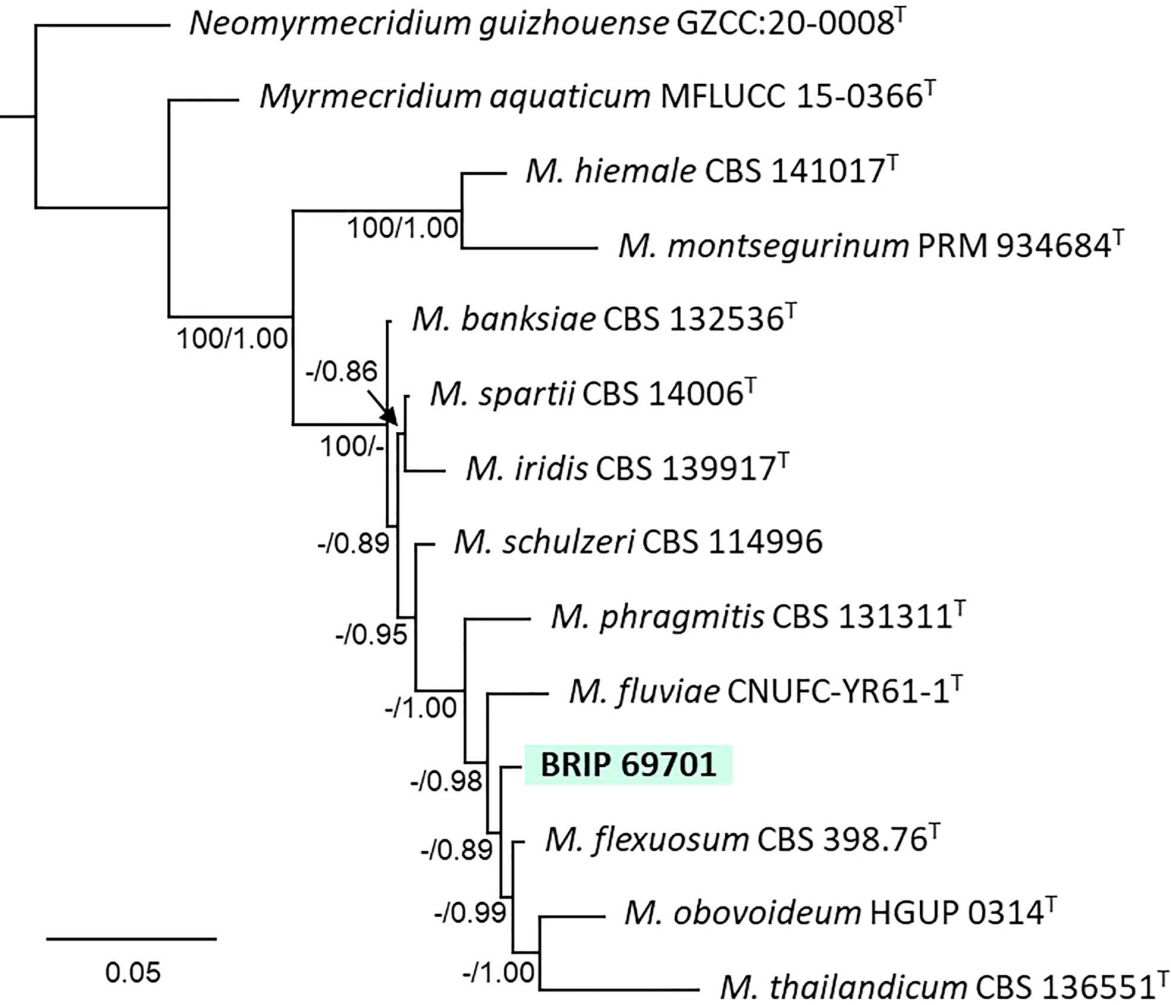
Phylogenetic tree inferred from a RAxML analysis based on a concatenated alignment of ITS, LSU, SSU and *rpb2* sequences of related species in Myrmecridiaceae (Myrmecridiales). RAxML bootstrap (bs) values greater than 70% and Bayesian posterior probabilities (pp) greater than 0.8 are given at the nodes (bs/pp). In bold font are the isolates from this study, and ex-type strains are indicated with ^T^.

The phylogenetic analysis of four gene regions (ITS, LSU, SSU, and *rpb2*) identified one isolate (BRIP 69701) as a novel species of *Myrmecridium* (Myrmecridiaceae; **Figure 13**).

#### 3.1.11 Trichosphaeriales

**Figure 14:**
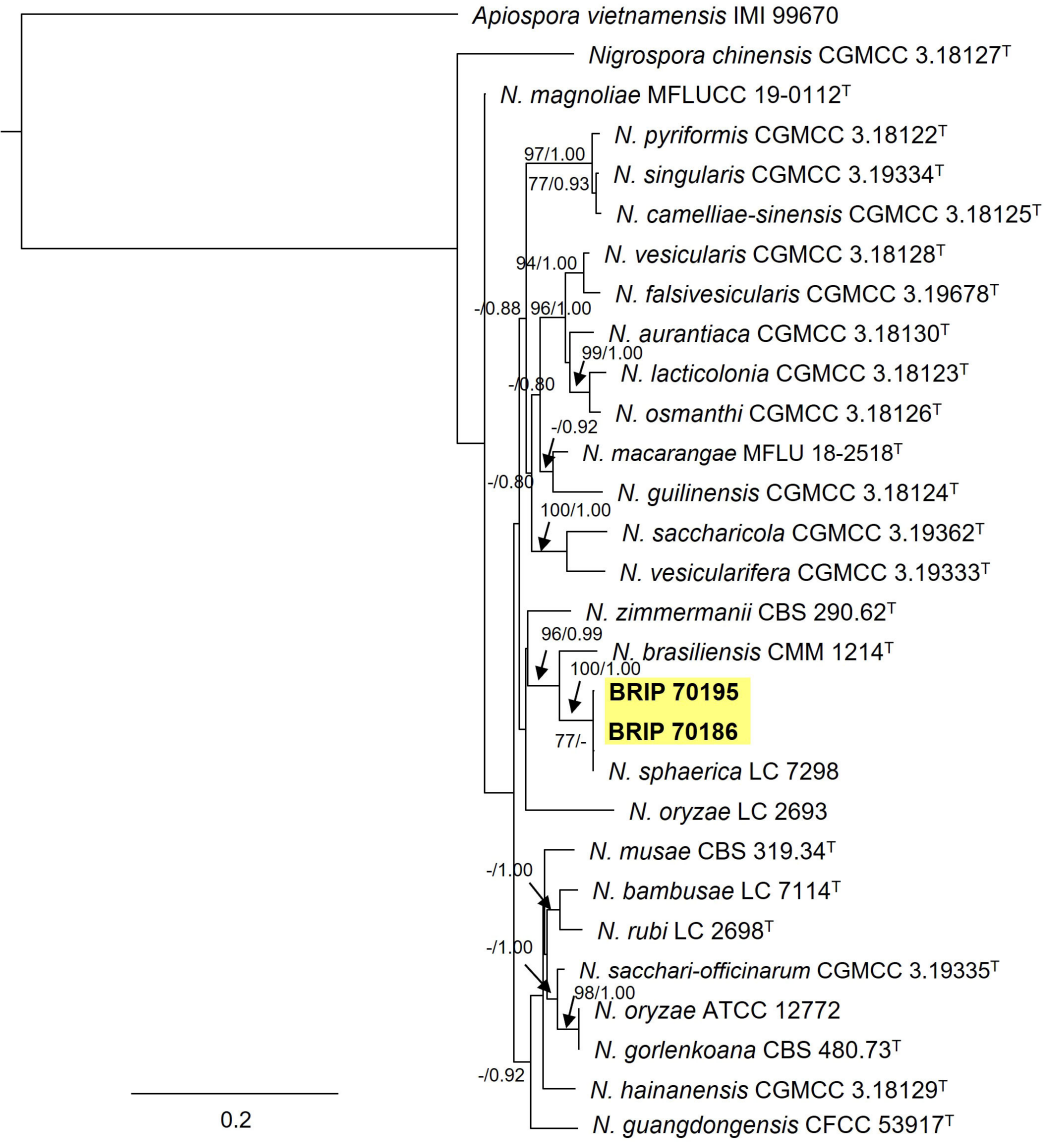
Phylogenetic tree inferred from a RAxML analysis based on a concatenated alignment of ITS, LSU and *tef1-α* sequences of related species in Trichosphaeriaceae (Trichosphaeriales). RAxML bootstrap (bs) values greater than 70% and Bayesian posterior probabilities (pp) greater than 0.8 are given at the nodes (bs/pp). In bold font are the isolates from this study, and ex-type strains are indicated with ^T^.

Two Trichosphaeriaceae isolates (BRIP 70186 and BRIP 70195) were identified using sequences from three gene regions (ITS, *tef1-α* and *tub2*; **Figure 14**) as the generalist plant pathogen *Nigrospora sphaerica* (Trichosphaeriaceae) (Wang et al., 2017; Hao et al., 2020).

#### 3.1.12 Xylariales

**Figure 15:**
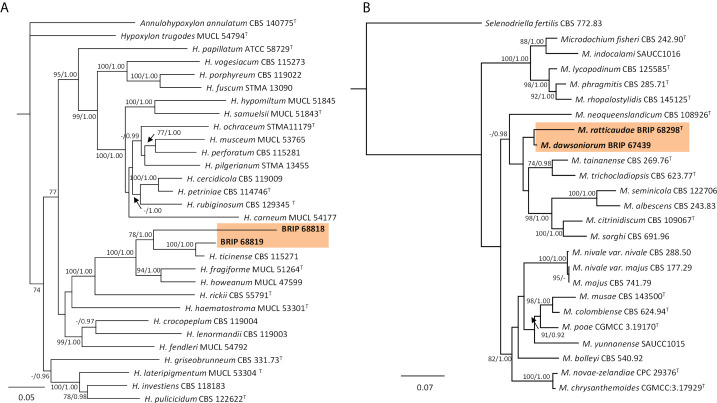
Phylogenetic trees inferred from RAxML analyses of strains in Xylariales based on concatenated alignments of **(A)** ITS, LSU, *rpb2* and *tef1-α* sequences of related species in Hypoxylaceae; and **(B)** ITS, LSU and *rpb2* sequences of related species in Microdochiaceae. RAxML bootstrap (bs) values greater than 70% and Bayesian posterior probabilities (pp) greater than 0.8 are given at the nodes (bs/pp). In bold font are the isolates from this study, and ex-type strains are indicated with ^T^.

The phylogenetic analysis of four gene regions (ITS, LSU, *rpb2*, and *tub2*) identified two isolates (BRIP 68818 and BRIP 68819) as two novel species of *Hypoxylon* (Hypoxylaceae; **Figure 15A**). The phylogenetic analysis of three gene regions (ITS, LSU, and *rpb2*; **Figure 15B**) identified three isolates to belong to *Microdochium* (Microdochiaceae). One isolate (BRIP 65649) represents the holotype of *M. dawsoniorum* (Crous et al., 2020), and the other (BRIP 68298) represent the holotype of *M. ratticaudae* (Crous et al., 2021).

## 4 Discussion

Grass-associated endophytes have been used for the biocontrol of insects and weeds in various agricultural and environmental systems. *Neotyphodium* spp. (Clavicipitaceae) has been applied to tall fescue (*Festuca arundinacea*) and perennial ryegrass (*Lolium perenne*) for the biocontrol of herbivorous insect pests, e.g., the root aphid *Aploneura lentisci* and wheat sheath miner *Cerodontha australis* (Saikkonen et al., 2006; Young et al., 2013). Other entomopathogenic fungi, including *Beauveria* (Cordycipitaceae; Mascarin and Jaronski, 2016), *Parametarhizium* (Clavicipitaceae; Gao et al., 2021) and *Clonostachys* (Bionectriaceae; Vega et al., 2008) have been used successfully to control insect pests. The fungal pathogen, *Stagonospora convolvuli*, produces metabolites that are toxic to crop pathogens (Boss et al., 2007a) and weeds (Boss et al., 2007b). There is evidence that endophyte-containing pasture grasses are more competitive against invading weeds (Saikkonen et al., 2013). Several fungal pathogens, including *Colletotrichum* (Glomerellaceae), *Alternaria* (Pleosporaceae), and *Phoma* (Didymellaceae), have been tested as bioherbicides for the control of target weeds (Morin, 2020). Currently in Australia, there is only one commercially registered product, Di-Bak® Parkinsonia, which contains three species, *Lasiodiplodia pseudotheobromae*, *Macrophomina phaseolina* and *Neoscytalidium novaehollandiae* in the Botryosphaeriaceae, that is available for the biocontrol of the invasive legume *Parkinsonia aculeata* (Galea, 2021). As individual plants may host hundreds of fungal species, finding a potential biocontrol agent among them is like searching for a needle in a haystack (Peay et al., 2016; Sutton, 2019).

In this study, we resolved the identities of 79 isolates of microfungi from *S. indicus* species across the state of Queensland. These microfungi were identified based on multi-locus sequence analysis, which is faster and arguably more accurate than using morphology alone, especially when taxonomic characters are lacking, unavailable or uninformative, and where taxonomic novelty is encountered.

Several fungi, including *Microdochium dawsonorium*, *Pestalotiopsis etonensis*, and *Neopestalotiopsis nebuloides*, have been recently described from *Sporobolus* spp. in Australia (Crous et al., 2020; Crous et al., 2021; Crous et al., 2022). These three fungal species were shown not to be pathogenic or host-specific against WGS (Lock, 2018; Kukuntod, 2020). Consequently, other isolates of Microdochiaceae and Pestalotiopsidaceae found in this study will likely not be prioritised for pathogenicity and host specificity testing. In contrast, *Alternaria*, *Bipolaris*, *Colletotrichum*, *Curvularia*, *Epicoccum*, and *Stagonospora* isolates identified in this study will be prioritised for pathogenicity studies, as these genera have been identified in biocontrol research or as grass pathogens (Luttrell, 1976; Bowers, 1986; Hetherington and Irwin, 1999; Bewick et al., 2000; Boss et al., 2007b), and are particularly diverse on grasses in Australia (Tan et al., 2016; Hernández-Restrepo et al., 2018; Tan et al., 2018).

Metagenomic approaches reveal a greater diversity of fungal taxa than isolation methods (Peay, 2014; Peršoh, 2015; Dissanayake et al., 2018). In our study, we have captured living fungal isolates from symptomatic tissues of grasses in the *S. indicus* complex. Many of these fungi are likely endophytes or opportunists. Our approach has identified these 79 living fungal isolates so they can now be prioritised for potential pathogenicity and host-specificity studies on *Sporobolus* spp. Many of these isolates are fast growing in the laboratory, which is a benefit for both *in vitro* and *in vivo* studies, e.g., it allows the mass-production of potential biocontrol agents at low cost thus ensuring the easy and quick production of a mycoherbicide. We acknowledge that our 79 isolates represent a small fraction of the total diversity associated with grasses in the *S. indicus* complex (Arnold et al., 2007; Higgins et al., 2011; Depetris et al., 2020).

In this study, we have shown that there is a high diversity of potentially pathogenic micro-fungal species associated with grasses in the *S. indicus* complex in Australia. Our method for finding potential biological control agents was to target infected plant tissue from coexisting and phylogenetically closely related grasses; isolate fungi from diseased leaf samples; and identify the resulting fungal species using multi-locus sequence analysis. This systematic approach may have application in many other agricultural and environmental systems.

## Data availability statement

The data presented in the study are deposited in NCBI's GenBank database. The accession numbers can be found in the **Supplementary Material**.

## Author contributions

JV is the project manager. JV and DH visited sites and looked for/collected samples. DH did the isolating, morphological ID, herbarium submission and culture maintenance. TS did the DNA extractions, PCR, Sequencing, data collation, manuscript outline and the phylogenetic analyses. YT provided advice and critique of the phylogenetic analyses, mycology, taxonomy, and manuscript development, as well as mentoring TS throughout. All authors provided feedback and critique of the various iterations of this manuscript.

## Funding

This project was supported by AgriFutures Australia (Rural Industries Research and Development Corporation), through funding from the Australian Government Department of Agriculture, Water and the Environment, as part of its Rural Research and Development for Profit program (RnD4Profit-18-04-014; PRJ-012376). Author TS was also supported by funding from the Queensland Government’s Advanced Queensland Women’s Research Assistance Program (WRAP049-2019D1).

## Acknowledgments

We acknowledge the Traditional Custodians of the land on which we work and sample in Australia, and their Elders past, present and emerging. Thanks to Drew Rapley and Nontaporn Kukuntod for technical support; and to Gavin Hunter and Roger Shivas for comments which greatly improved this manuscript.

## Conflict of interest

The authors declare that the research was conducted in the absence of any commercial or financial relationships that could be construed as a potential conflict of interest.

## Publisher’s note

All claims expressed in this article are solely those of the authors and do not necessarily represent those of their affiliated organizations, or those of the publisher, the editors and the reviewers. Any product that may be evaluated in this article, or claim that may be made by its manufacturer, is not guaranteed or endorsed by the publisher.
